# Machine and deep learning in inflammatory bowel disease

**DOI:** 10.1097/MOG.0000000000000945

**Published:** 2023-05-08

**Authors:** Fatima Zulqarnain, S. Fisher Rhoads, Sana Syed

**Affiliations:** Department of Pediatrics, University of Virginia School of Medicine, Charlottesville, Virginia, USA

**Keywords:** artificial intelligence, deep learning, inflammatory bowel disease, machine learning

## Abstract

The Management of inflammatory bowel disease (IBD) has evolved with the introduction and widespread adoption of biologic agents; however, the advent of artificial intelligence technologies like machine learning and deep learning presents another watershed moment in IBD treatment. Interest in these methods in IBD research has increased over the past 10 years, and they offer a promising path to better clinical outcomes for IBD patients.

**Recent findings:**

Developing new tools to evaluate IBD and inform clinical management is challenging because of the expansive volume of data and requisite manual interpretation of data. Recently, machine and deep learning models have been used to streamline diagnosis and evaluation of IBD by automating review of data from several diagnostic modalities with high accuracy. These methods decrease the amount of time that clinicians spend manually reviewing data to formulate an assessment.

**Summary:**

Interest in machine and deep learning is increasing in medicine, and these methods are poised to revolutionize the way that we treat IBD. Here, we highlight the recent advances in using these technologies to evaluate IBD and discuss the ways that they can be leveraged to improve clinical outcomes.

## INTRODUCTION

**FIGURE 1:**
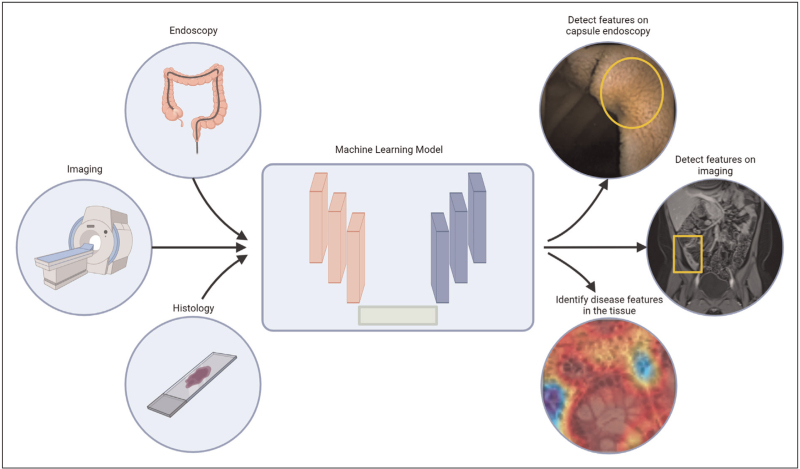
Machine learning models allow for the identification of disease features. Machine learning models can use data from endoscopy, imaging, and histology to detect lesions, identify strictures, and identify histological features of disease.

Inflammatory bowel disease (IBD) is characterized by chronic intestinal inflammation with periods of remission and relapse [1] and categorized as ulcerative colitis, Crohn's disease, or indeterminate colitis based on a combination of clinical, endoscopic, and histologic data [1,2]. Currently, the gold standard for IBD diagnosis is ileocolonoscopy and esophagogastroduodenoscopy (EGD) combined with histology analysis [3]; however, novel machine and deep learning technologies present promising new avenues to significantly enhance our understanding of IBD and optimize remission standards to aim for better patient outcomes. Additional diagnostic modalities, including capsule endoscopy, magnetic resonance enterography, and computed tomography enterography are becoming more common in aiding diagnosis and evaluation [4,5], and the data produced from these techniques can be analyzed by machine and deep learning models to inform management of disease (Fig. 1). Although our understanding of the cause of IBD is still evolving [6], our treatment paradigms have markedly changed over the past decade, as biologic agents have become more prevalent [7], and machine and deep learning technologies are uniquely poised to revolutionize our approach to IBD treatment again. 

**Box 1:**
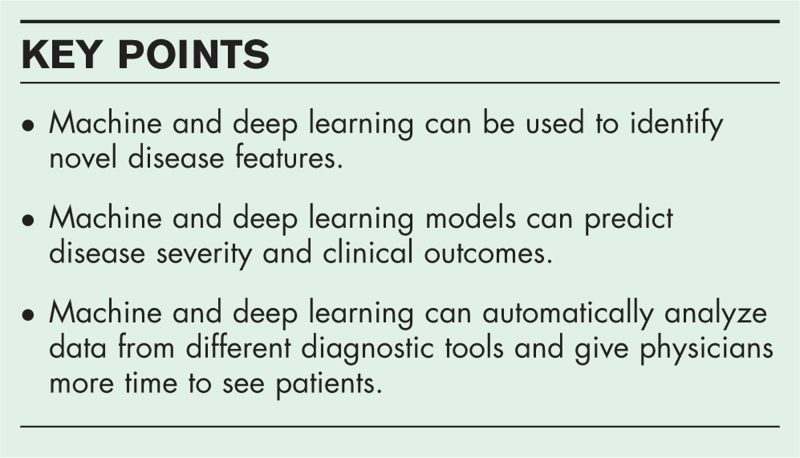
no caption available

Artificial intelligence refers to computing methods capable of completing tasks that traditionally require human intelligence [8]. A summary of artificial intelligence concepts can be found in Table 1. To date, the majority of artificial intelligence technologies in medicine have been assistive, although the Food and Drug Administration (FDA) has approved a fully autonomous system to detect diabetic retinopathy [9]. Past use of computers to aid diagnosis and management of disease has relied on analyzing data using a concrete set of prescribed rules; however, novel machine learning methods allow researchers to ‘train’ or ‘teach’ computer models iteratively by exposing them to larger datasets annotated with clinical findings and disease outcomes [10]. Over time, the machine ‘learn’ patterns in the data and can predict specific outcomes from new data inputs. Assistive artificial intelligence was embraced in studying nongastrointestinal diseases like breast cancer [11–14], and interest in artificial intelligence in gastroenterology has increased over time. The number of publications displayed using a keyword search on PubMed of ‘artificial intelligence’ and ‘gastroenterology’ has increased from just 10 references in 2012 to more than 600 in 2022. Artificial intelligence technologies are particularly well suited to study IBD because diagnosis and management requires the synthesis of large, heterogeneous data such as endoscopic evaluation, radiologic evaluation, and histopathologic evaluation. These techniques can be leveraged to identify the mathematical functions that associate these different types of data, allowing for the integration of multiple diagnostic modalities into a unified model to evaluate disease. Furthermore, use of artificial intelligence may significantly improve clinical workflow by reducing the immense amount of time spent in interpreting data, allowing clinicians to spend more time interacting face-to-face with patients, leading to improved data collection and patient outcomes.

**Table 1:** Definitions of artificial intelligence concepts

Concept	Definition
Artificial intelligence	Artificial intelligence refers to computational methods through which computers are able to complete activities that traditionally required human intelligence to complete
Machine learning	Artificial intelligence systems that allow a model or system to learn and improve iteratively as it is exposed to additional data
Deep learning	Machine learning models that are created using complex algorithms that are inspired by the organization of the human brain with many discrete nodes or ‘neurons’ and can identify important patterns or features in a dataset
Autonomous artificial intelligence	Autonomous artificial intelligence refers to when a system is making predictions and decisions without any real-time human input, for example, self-driving cars
Assistive artificial intelligence	Assistive artificial intelligence systems are designed to augment a human operator's ability to perform a certain task
Convolutional neural network	Convolutional neural networks are a type of deep learning model that uses a series of convolutional layers containing filters to detect patterns of increasing complexity in an input image as the data runs through the model

## MACHINE LEARNING IN ENDOSCOPY

The gold-standard for IBD diagnosis is thorough examination of patient-derived biopsies collected during ileocolonoscopy and esophagogastroduodenoscopy. In addition to informing diagnoses, gross examination of the intubated bowel also provides important contextual information about the extent, continuity, and severity of inflammation. Unfortunately, current diagnostic methods cannot definitively classify some patients’ disease following initial examination. Establishing the correct diagnosis as early as possible is crucial because the treatment for ulcerative colitis and Crohn's disease can vary – especially in severe cases, where surgical interventions are considered. In order to address this challenge, Chierici *et al.*[15] developed a deep learning model to distinguish healthy versus inflamed tissue, ulcerative colitis from healthy tissue, and ulcerative colitis from Crohn's disease. Although the model's performance was best in the first two tasks, it was able to distinguish ulcerative colitis from Crohn's disease fairly accurately with a Matthew's correlation coefficient of 0.688. The results of their proof-of-concept study demonstrate that, with further investigation, a deep learning model capable of distinguishing ulcerative colitis from Crohn's disease could be a powerful tool for clinicians when diagnosing IBD. Although further work in the specific domain of diagnosis is necessary, there has been a surge in interest in leveraging artificial intelligence to assist in characterizing severity of IBD, predicting clinical outcomes, and identifying lesions using endoscopy data.

As target treatment outcomes have evolved over time for IBD, several indices for scoring the severity of inflammation on endoscopy have been developed [16–20]; however, these metrics have not been widely adopted and require clinicians to manually calculate the scores [21]. Several studies have investigated the use of machine learning models to score bowel inflammation severity – particularly in ulcerative colitis – using traditional endoscopy images and video as well as capsule endoscopy footage [22,23,24^▪^,^25^]. In one such study, Iacucci *et al.* developed a model, which predicted endoscopic and histologic remission in ulcerative colitis patients using white light endoscopy and virtual chromoendoscopy video data [23]. Separately, Tekenaka *et al.* created a deep learning model that can be implemented in real-time during endoscopy to predict histological remission and calculate an ulcerative colitis endoscopic index of severity score (UCEIS) [16,24^▪^]. Their model's histologic prediction agreed with pathologists’ assessment 97% of the time, demonstrating the model's ability to accurately assess inflammation on endoscopy.

In addition to grading inflammation severity, machine learning is currently being used to predict clinical outcomes based on endoscopy data. Maeda, *et al.* used an artificial intelligence system called EndoBRAIN-UC and endocytoscopy [26^▪▪^], a technique involving the use of a microscope attached to the endoscope, to predict the likelihood of active disease or healing in patients undergoing colonoscopy. They found that for patients with a prediction of active disease, the risk of clinical relapse over 12 months was 28.4% versus only 4.9% in patients with predictions of healing, highlighting the power of artificial intelligence tools to predict clinical outcomes and inform clinical management of IBD.

Lastly, there have been significant advances in utilizing machine learning models to identify ulcers and other mucosal features using capsule endoscopy data [27]. Ferreira *et al.*[28] developed a model that automatically detects ulcers in the small bowel capsule endoscopy data, whereas Afonso *et al.*[29] further developed the model to automatically quantify ulcers and predict their hemorrhagic potential. Furthermore, the model was able to analyze the footage in 16 min on average, whereas the same video could take a clinician up to 120 min to review. With adoption of this technology, the time spent reviewing capsule endoscopy footage could be spent seeing more patients.

## MACHINE LEARNING IN RADIOLOGY

In addition to ileocolonoscopy and biopsy review, additional radiologic modalities are increasingly used to diagnose and characterize IBD, especially when small bowel inflammation is suspected. Researchers have investigated numerous metrics to assess intestinal inflammation and stricture using MRE data [30–32]; however, machine and deep learning are currently being explored as a mechanism to identify features associated with disease and aid diagnosis. The development of such indices for evaluation of IBD is paramount; however, development of these indices is hindered by relying on time-consuming, manual review of MRE data by researchers. machine learning techniques allow for partial or potentially full automation of this review process. Ziselman *et al.* trained a model with residual connections on annotated T2-weighted MRE scans from the ImageKids study, which accurately segmented MRE images of the terminal ileum and classified images as having normal or increased bowel wall thickness [33–35]. Notably, their model was trained on data that can be derived from MRE without intravenously administered contrast, increasing accessibility to this technique. Similarly, Arkko *et al.* developed a machine learning method to detect Crohn's disease using quantified motility measurements from MRE data, which performed well in the dataset with high-pixel resolution, low temporal resolution, and high temporal length [36]. However, their model's performance deteriorated by about 30% in the other two datasets tested, highlighting that further investigation is needed to validate this approach.

Computed tomography enterography (CTE) is less commonly used in the evaluation of IBD because of its requisite radiation exposure; however, there have been similar advances using machine learning for this diagnostic method. A deep learning model was developed and demonstrated better performance than human radiologists in assessing bowel fibrosis [37^▪^]. Additionally, researchers developed a convolutional neural network (CNN) to identify differences in volumetric visceral adipose tissue content visualized on CTE in patients with ulcerative colitis and Crohn's disease, which found that visceral adipose tissue phenotype differed between the two groups [38], demonstrating that CNN-based machine learning models can extract features from data that may inform diagnosis of IBD in the future.

Lastly, Guez *et al.* developed a multimodal random forest model that integrated MRE data with normalization of biochemical markers including C-reactive protein and fecal calprotectin, and they found that their fusion model could more accurately predict Crohn's disease than a linear regression model based on MaRIA [39,40]. This study exemplifies how machine and deep learning models can integrate multiple types to provide more accurate predictions than traditional statistical methods.

## MACHINE LEARNING IN HISTOLOGY

Although mucosal healing is the primary target for the treatment of IBD, histologic remission may become an important and reasonable objective in the near future. The use of artificial intelligence can optimize these efforts using a multipronged approach: automate histopathologic scoring using existing scoring systems, extract meaningful histologic features of disease, and/or develop new metrics of disease activity.

Many histologic scoring indices have been developed for categorizing ulcerative colitis over the years. The Nancy index (NI) [41], was first published in 2017. Using a dataset of 200 images in patients with ulcerative colitis, a novel artificial intelligence system was developed in Nancy, France, to automate the assessment of the Nancy index. The average intra-class coefficient (ICC) among the three human annotators was 0.89, and remarkably, the average ICC between the histopathologists and the artificial intelligence tool was 0.87, illustrating the potential for using artificial intelligence to standardize histological scoring [42^▪^].

Histologic remission in ulcerative colitis, defined by the European Crohn's and Colitis Organisation (ECCO) as the absence of intraepithelial neutrophils, erosions, and ulcerations [43], has become an important objective of therapy. The Paddington International virtual ChromoendoScopy ScOre (PICaSSO) Histologic Remission Index (PHRI) was developed using biopsies from 307 patients across 11 centers in Europe and North America [18]. The study team wanted to develop a simple histologic index that correlated with published histology scores [41,44,45], endoscopy activity scores [16,46], clinical outcomes, and was conducive to automating scoring using artificial intelligence by focusing on a single-variable neutrophil infiltration. Since developing the PHRI, it has been used to develop multiple artificial intelligence algorithms. Gui *et al.*[47^▪▪^] developed a CNN-based model to detect neutrophils in whole slide images and subsequently classify images into either a histologic remission or nonremission class. Villanacci *et al.*[48] also developed a semi-supervised transfer learning model using images with pixel-level neutrophil annotations from patients with ulcerative colitis to predict the presence of neutrophils. Subsequently, a second model combined the features of all patches in each biopsy to classify the presence or absence of ulcerative colitis with high accuracy. These results demonstrate that designing new metrics that can easily be incorporated into digital pathology artificial intelligence systems to interpret histopathology data and inform clinical management of IBD.

Machine learning can also be used to extract meaningful histologic features of disease. Deep learning has previously been used to analyze intestinal histopathologic images of surgical specimens in patients with Crohn's disease, showing adipocyte shrinkage and mast cell infiltration to predict postoperative recurrence of Crohn's disease within 2 years after surgery [49^▪^]. Similarly, mucin depletion is a potential histologic indicator of clinical relapse in ulcerative colitis [50]; however, low inter-observer concordance limits its utility as a predictor. Recently, a deep learning model was trained to quantify goblet cell mucus area in whole slide images from patients with ulcerative colitis in endoscopic remission [51^▪^], and they found that patients with a greater ratio of goblet cell mucus area to the epithelial cell and goblet cell mucus area had a lower relapse rate over 12 months, demonstrating that deep learning models can help to standardize interpretation of image data. Furthermore, researchers developed a deep-learning model to identify eosinophils in tissue from ulcerative colitis patients and quantify eosinophil density [52]. From these calculations, they observed that patients on corticosteroid therapy had significantly lower eosinophil density, demonstrating the capability of deep learning models to identify unique disease features using histopathology images. However, there is still a gap in the literature on using machine learning-based histopathologic analysis methods to search for histologic features of dysplasia resulting from or comorbid with IBD.

## CONSIDERATIONS FOR USING ARTIFICIAL INTELLIGENCE IN CLINICAL PRACTICE

**FIGURE 2:**
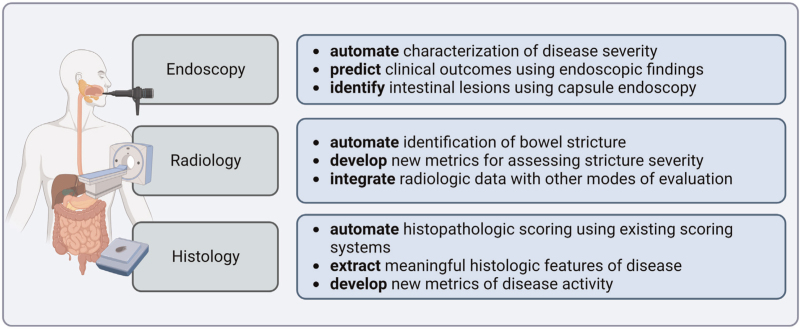
Summary of machine learning capabilities in inflammatory bowel disease.

Despite artificial intelligence's potential to revolutionize medical practice, several formidable obstacles must be addressed prior to the widespread implementation of artificial intelligence in the clinical setting. Although artificial intelligence is optimally suited to parse the wealth of modern clinical information and find novel insights in IBD (Fig. 2), training and deployment of these models are significantly hindered by the critical lack of large, publicly available, pathologist-annotated datasets, the lack of data sharing, and the relative rarity of IBD. Furthermore, the controls used for comparison are often tissue with no overt signs of inflammation or tissue obtained from participants with other gastrointestinal diseases. Thus, no clear reference dataset exists for ‘what is normal’. The creation of such reference datasets is necessary to allow for extensive testing of artificial intelligence systems so that they can be leveraged to achieve better clinical outcomes. Another limitation of using artificial intelligence comes from having insufficient or inconsistent datasets to use for modeling. When models are trained on a single dataset from one site, the images are often consistent because of standardized tissue staining and slide digitization. Importantly, there may be considerable image differences when using images from other sites limiting the generalizability of models.

Because of the promise of decreasing human error, artificial intelligence has been lauded as an objective means of assisting in various areas of clinical practice, including disease diagnosis, prevention, monitoring, therapy, and long-term management. Along with immense promise, the use of artificial intelligence in medical practice comes with the necessity to consider the ethical principles of medicine: justice, beneficence, nonmaleficence, and autonomy. As the improper use of artificial intelligence can potentially violate all three of these principles, researchers and doctors must hold each other accountable while creating and using these technologies. The use of artificial intelligence without extensive evidence of improved patient outcomes would violate nonmaleficence, so it is crucial to have a ‘human in the loop’ to oversee the implementation of and decisions of artificial intelligence systems to ensure that they are not causing harm to patients. Additionally, using datasets that are not expansive and diverse can lead to bias in algorithms that could affect how certain groups receive care. Lastly, the implementation of artificial intelligence in clinical practice without informed consent from a patient would violate their autonomy [53,54]. Machine and deep learning methods have the potential to transform the way that physicians diagnose and manage IBD drastically; however, significant care must be taken in developing and implementing these technologies to ensure that they help achieve better clinical outcomes.

## CONCLUSION

Machine and deep learning models are becoming increasingly common in medicine. Although further studies are necessary before implementing these technologies in a clinical setting, researchers have demonstrated their utility in aiding the endoscopic, radiologic, and histologic evaluation of IBD. Although there are obstacles to implementing these models in the clinical setting, we will likely see these technologies develop and seek regulatory approval for clinical use in the near future.

## Acknowledgements

*We would like to thank Adam Greene, PhD, for his editorial insight during the preparation of our manuscript. Figure 1 and 2 were created using Biorender.com.*

### Financial support and sponsorship

*Research reported in this publication was supported by National Institute of Diabetes and Digestive and Kidney Diseases (NIDDK) of the National Institutes of Health under award number K23DK117061-01A1 (S.S.) and award number R01DK131491 (S.S.).*

### Conflicts of interest

*There are no conflicts of interest.*

## REFERENCES AND RECOMMENDED READING

Papers of particular interest, published within the annual period of review, have been highlighted as:

▪ of special interest
▪▪ of outstanding interest
